# CO_2_ Conversion to Alcohols over Cu/ZnO Catalysts: Prospective Synergies between Electrocatalytic and Thermocatalytic Routes

**DOI:** 10.1021/acsami.1c15871

**Published:** 2021-12-29

**Authors:** Hilmar Guzmán, Fabio Salomone, Samir Bensaid, Micaela Castellino, Nunzio Russo, Simelys Hernández

**Affiliations:** † CREST Group, Department of Applied Science and Technology (DISAT), Politecnico di Torino, C.so Duca degli Abruzzi, 24, 10129 Turin, Italy; ‡ IIT—Istituto Italiano di Tecnologia, Via Livorno, 60, 10144 Turin, Italy

**Keywords:** Cu/ZnO, CO_2_ conversion, CO dimerization, C_2+_ products, alcohols

## Abstract

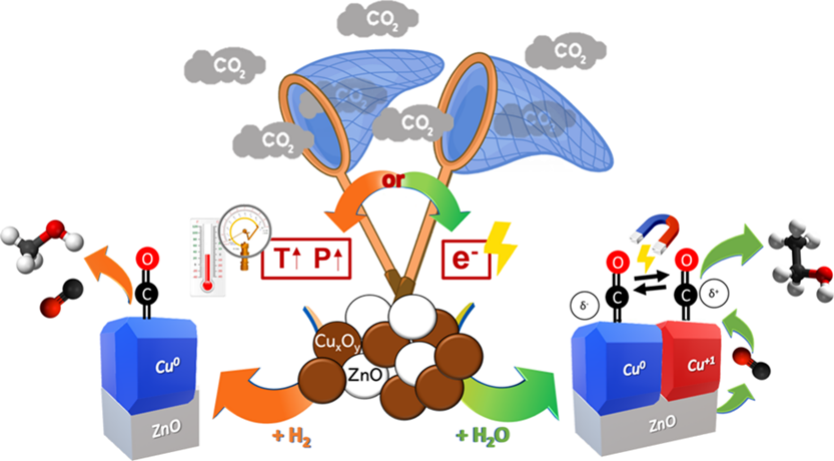

The development of efficient catalysts is one of the main challenges in CO_2_ conversion to valuable chemicals and fuels. Herein, inspired by the knowledge of the thermocatalytic (TC) processes, Cu/ZnO and bare Cu catalysts enriched with Cu^+1^ were studied to convert CO_2_ via the electrocatalytic (EC) pathway. Integrating Cu with ZnO (a CO-generation catalyst) is a strategy explored in the EC CO_2_ reduction to reduce the kinetic barrier and enhance C–C coupling to obtain C_2+_ chemicals and energy carriers. Herein, ethanol was produced with the Cu/ZnO catalyst, reaching a productivity of about 5.27 mmol·g_cat_^–1^·h^–1^ in a liquid-phase configuration at ambient conditions. In contrast, bare copper preferentially produced C_1_ products like formate and methanol. During CO_2_ hydrogenation, a methanol selectivity close to 100% was achieved with the Cu/ZnO catalysts at 200 °C, a value that decreased at higher temperatures (i.e., 23% at 300 °C) because of thermodynamic limitations. The methanol productivity increased to approximately 1.4 mmol·g_cat_^–1^·h^–1^ at 300 °C. Ex situ characterizations after testing confirmed the potential of adding ZnO in Cu-based materials to stabilize the Cu^1+^/Cu^0^ interface at the electrocatalyst surface because of Zn and O enrichment by an amorphous zinc oxide matrix; while in the TC process, Cu^0^ and crystalline ZnO prevailed under CO_2_ hydrogenation conditions. It is envisioned that the lower *CO binding energy at the Cu^0^ catalyst surface in the TC process than in the Cu^1+^ present in the EC one leads to preferential CO and methanol production in the TC system. Instead, our EC results revealed that an optimum local CO production at the ZnO surface in tandem with a high amount of superficial Cu^1+^ + Cu^0^ species induces ethanol formation by ensuring an appropriate local amount of *CO intermediates and their further dimerization to generate C_2+_ products. Optimizing the ZnO loading on Cu is proposed to tune the catalyst surface properties and the formation of more reduced CO_2_ conversion products.

## 1 Introduction

Greenhouse gas emissions from natural systems and human activities have caused a shift in climate patterns. Carbon dioxide (CO_2_) is the key contributor to global climate change in the atmosphere. Climate change emerges because the Earth does not have enough capacity to neutralize all the emitted CO_2_, meaning that humanity is demanding more than the Earth can offer.^1^ Over the last century, the concentration of atmospheric CO_2_ has increased (reaching 417 ppm in 2020). For this reason, the synthesis of high added-value products, for example, alcohols by CO_2_ conversion, is a promising approach to mitigate climate change.^2^ However, it represents a major challenge because CO_2_ is a thermodynamically stable molecule. It entails multielectron-transfer reactions and parallel reaction mechanisms, the main causes of low selectivity and productivity.

The hydrogenation of CO_2_ to value-added products can mitigate its emission into the atmosphere:^3^ it can be used to produce commodities employed as fuels or feedstock to generate numerous energy-dense chemicals using well-established processes. Such chemical recycling can be achieved by electrocatalytic (EC) CO_2_ reduction (CO_2_R)^4−6^ and thermocatalytic (TC) CO_2_R.^7−9^ The first can be coupled with a renewable electricity source and carried out under mild reaction conditions, using water for the in situ generation of protons (H^+^). Instead, thermochemical conversion is conducted in more severe reaction conditions of pressure (≥2 MPa H_2_) and temperature (≥220 °C). In the latter, H_2_ could be supplied by water electrolysis using renewable energy to be sustainable. However, in both cases, the catalyst is the main challenge; in fact, it plays a crucial role in determining the activity and selectivity of the CO_2_ conversion process.^10,11^

To date, the most competitive performance in electrocatalytic systems has been achieved for CO (or syngas) production on Ag-based^12^ and Au-based^13^ catalysts, reaching high Faradaic efficiencies (FE > 70%) and relevant current densities (>50 mA cm^–2^).^14−16^ From a techno-economic point of view, formate production is another cost-effective product.^17^ In fact, Avantium recently patented a bismuth-indium electrocatalyst for the formate production from the electrochemical CO_2_R, producing FE = 95% up to 200 mA cm^–2^.^18^ On the other hand, the performance of Cu-based electrocatalysts is among the best ones that have ever been achieved to transform CO_2_ into C_1+_ products.^2,19,20^ In particular, C_2+_ oxygenate products like alcohols are attractive because they have a high volumetric energy density, compatible with the current energy infrastructure, and can be stored as liquids at room conditions.^2^ According to this, scientific researchers have achieved relevant quantities of methanol (MeOH), ethanol (EtOH), and *n*-propanol (*n*-PrOH) on Cu-based electrocatalysts.^4,21−24^ However, these reduction products will be economically viable if high production rates are also attained.^17^

In the case of the TC CO_2_ reduction process, the commercial implementation of CO_2_ hydrogenation into C_2+_ oxygenates compounds has not yet been reached.^2,3^ However, some reports in the literature show future opportunities using Cu-, Fe-, and Co-based catalysts.^25,26^ Nonetheless, enhancing this process remains an ongoing challenge because of the high C–C coupling barriers.^27,28^ The single metal (e.g. Cu) is not very active by itself. For this reason, amphoteric metal oxides (i.e., ZnO and ZrO_2_) have been investigated as metal supports. In this regard, Cu/ZnO composites are active catalysts widely used for CO_2_ hydrogenation to methanol.^29,30^ It has been shown that the metal/metal oxide interface and the synergistic effect of different phases on the catalysts control their selectivity and performance.^31^ This catalytic strategy seems to be also suitable for the CO_2_ co-electrolysis to C_2+_ products. It involves increasing the local concentration of the *CO intermediate by integrating Cu with another CO-generation catalyst (e.g., ZnO and ZrO_2_). Concerning this, Munir et al.^32^ have evidenced liquid products such as methanol, formate, n-propanol, and acetone on a Cu/ZnO electrode, reaching a high FE of approximately 97%. They attributed the C–C coupling to the Cu–Zn interface and the formation of Cu^0^ sites rather than Cu^1+^ after electrochemical reduction. Andrews et al.^33^ have stated that the natural interfaces of Cu and ZnO could lower the barriers for the hydrogenation of adsorbed CO for producing methanol and ethanol and trace levels of propanol. In fact, they increased the FE of methanol by approximately 10-fold and the FE of ethanol by approximately 27-fold when Cu/ZnO electrodes are used in place of the Cu bare catalyst.

Likewise, Albo et al.^5,34,35^ have observed that Cu_2_O/ZnO catalysts enhance selectivity to methanol and ethanol and have high stability in CO_2_ reduction. The cooperation of these two metals (Cu and Zn) and carbon materials has also been investigated for electrochemical CO_2_R. Geioushy et al.^36^ have synthesized graphene/ZnO/Cu_2_O hybrid materials, and *n*-propanol was the only liquid product detected during the reaction. The FE of *n*-propanol was found to be 30% on this catalyst. The C–C–C formation has been ascribed to the cooperation of these three components. In a recent work, Zhang et al.^37^ have designed a Cu/ZnO tandem electrode by adding a layer of ZnO on top of a Cu catalyst. It increases the efficiency of *CO intermediate utilization and, therefore, the FE of C_2+_ products by approximately 1.2-fold compared to the bare Cu electrode.

CuZn-based materials are promising CO_2_ reduction catalysts for alcohol production, considering their low cost and high abundance. Herein, ZnO and Cu nanoparticles were used as intermediate *CO- and C–C coupling selective materials, respectively. The mixture Cu/ZnO material performance was compared with a bare Cu electrode. This strategy is inspired by the knowledge of the TC CO_2_R process, for which high methanol selectivity could be achieved at high *T* and *P* (240–280 °C and 20–80 bar_,_ respectively).^7,29,30^ The strategy consists of (i) enhancing the CO_2_ adsorption and reducing the barrier of the first up-hill reaction at the catalyst surface, leading to the production of *CO; (ii) tuning the adjacent chemical environment around the Cu atoms and the binding strengths of targeted intermediates using a stable metal oxide catalyst like ZnO, which is also selective to the CO formation,^38,39^ and (iii) promoting its subsequent coupling on the Cu-based catalyst surface.^40^

It is worth noting that although there are important differences between electrocatalysis and thermocatalysis (like the possible presence of an electrolyte solution, counterions, and electric fields in the first one), the reduction reaction can occur by following the same kinetic laws and similar mechanisms.^41^ Given the nature of the catalytic environment in thermocatalysis, more detailed characterization and theoretical simulations can be found in the literature. In this context, many of the fundamental constructs that govern gas-phase catalysis could also be integrated into electrocatalysis and help develop new electrocatalysts or/and effective conditions for the reaction. Thus, different from previous studies, herein, we tested the same catalysts for these two CO_2_ conversion technologies and performed an ex situ characterization of the tested materials by X-ray diffraction (XRD), field emission scanning electron microscopy (FESEM), and X-ray photoelectron microscopy (XPS) to find potential synergies for future developments.

## 2 Materials and Methods

### 2.1 Preparation of Cu and Cu/ZnO Electrodes

The Cu/ZnO (CZ) mixture catalysts were prepared using commercial copper and zinc oxide nanoparticles (NPs) (Sigma Aldrich). The copper nanoparticles were selected with a size range of 40–60 nm (Cu), while the zinc oxide was around 20–25 nm. The samples were prepared by the preoxidation of the Cu NPs at 150 °C for 2 h in static air (Cu calc) and then manually mixing it with ZnO (CZ calc). The molar ratio between Cu and ZnO is equal to 65/35. The electrodes were manufactured by depositing a homemade catalytic ink on a porous carbon support (Toray carbon paper, thickness 0.19 mm Teflon 20 (±5) wt % treated, Quintech) by dropping. The catalytic ink is composed of different components: (i) powder catalysts; (ii) Nafion (dispersion, 5 wt % in water and 1-propanol) (Sigma Aldrich) as the binder for the particles; (iii) 20% of multiwalled carbon nanotubes (MWCNT) (Sigma Aldrich) to improve dispersion and electron conductivity of the electrocatalyst; and (iv) isopropanol (99% of purity, Sigma Aldrich) for well dispersing all the components. A mass ratio of catalyst/Nafion of 70:30 and an isopropanol/solids mass ratio of 97:3 were used. The tests were performed with a catalyst loading of 1.5 mg cm^–2^. Each Cu-based electrode was prepared with a geometric area of 1 cm^2^. The deposition process was performed by placing the carbon paper on a heating plate at 120 °C to ensure complete solvent evaporation. All the electrodes were then kept on the heating plate for 15 min before their usage.

### 2.2 Characterization of the Catalysts

FESEM (ZEISS MERLIN), with an energy-dispersive X-ray spectroscopy (EDS) system, conducted at 3 kV, was employed to obtain the morphology and the content of the relative elements of the samples. The samples were prepared by dispersing a small quantity of the particles in isopropanol via ultrasonic mixing for 30 min. Successively, a dispersion drop was placed on a nickel grid coated with an amorphous carbon layer. Finally, the sample was dried at room temperature before the FESEM analysis.

The specific surface area evaluated according to the Brunauer–Emmett–Teller (BET) theory and the total pore volume were determined by measuring N_2_ adsorption/desorption isotherms at 77 K in a volumetric equipment TriStar II 3020 (Micromeritics). All the samples were outgassed at 200 °C for 2 h before the measurements. The Barrett–Joyner–Halenda (BJH) method was applied to determine the pore size distributions from experimental isotherms using the Kelvin model of pore filling.

The XRD technique was used to obtain information about the crystallinity of the samples using a diffractometer (Panalytical X’Pert PRO) working in Bragg–Brentano configuration and equipped with Cu Kα radiation (λ = 1.5418 Å) set at 40 kV and 40 mA. The Scherrer equation (*D = k*λ/βcosθ) was used to calculate the crystallite sizes of the powder catalysts. *D* is the average crystallite size (nm), *k* is the shape factor (0.90), λ is the wavelength of the X-ray radiation (0.15418 nm), and β is the full-width at half-maximum, which was corrected for instrumental broadening. XRD examined the powder samples in the 2θ range of 20–80° with a scanning step of 0.013°. After the tests, electrodes were examined in the 2θ range of 20–150° with a scanning step of 0.020°.

XPS measurements were performed using a PHI 5000 Versa Probe (Physical Electronics) system. The instrument has a monochromatic X-ray source of 1486.6 eV (Al K-alpha) for determining the surface composition of the prepared materials. All core-level peak energies were referenced to the C1s peak at 284.5 eV, and the background signal, in high-resolution (HR) spectra, was detracted by means of a Shirley function. The Multipak 9.7 software was used to complete the deconvolution procedure.

### 2.3 Electrocatalytic CO_2_ Reduction Tests

**Figure 1:**
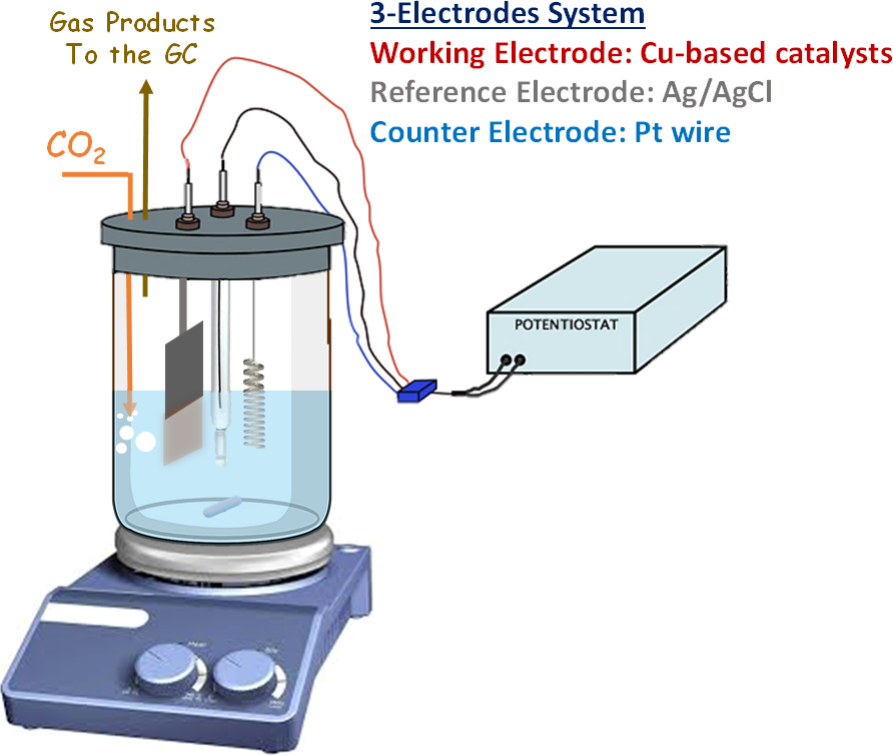
Simplified conceptual scheme of the electrochemical CO_2_ reduction setup.

The electrochemical characterization of the samples consists of testing the catalytic activity in a CO_2_-saturated 0.1 M KHCO_3_ solution (70 mL) using a traditional 3-electrode electrochemical cell at ambient conditions (see Figure 1). The cell was equipped with a platinum wire as a counter electrode and a silver/silver chloride electrode (Ag/AgCl, 3 M NaCl) as the reference electrode. The prepared Cu-based electrodes with a geometric area of 1 cm^2^ were used as working electrodes. A Biologic VSP-300 multichannel potentiostat was used to carry out the electrochemical tests.

Cyclic voltammetry (CV) was performed from 0.5 to −1.4 V vs RHE (at a scan rate of 30 mV s^–1^) to evaluate the electrochemical behavior of the prepared catalysts. Linear sweep voltammetry (LSV) was performed from 0.5 to −2.4 V vs RHE (at a scan rate of 5 mV s^–1^) to estimate the onset potential of the catalysts under CO_2_ bubbling into the electrolyte. CO_2_ coelectrolysis was carried out by performing a chronoamperometry (CA) at a constant potential for 2 h to determine the selectivity of each catalyst material. The CO_2_ flow rate was set via a mass flow controller (EL-Flow Select, PN64) at 8.86 N mL min^–1^.

The concentration of gaseous products was determined by using an online gas chromatograph (Inficon—Micro GC Fusion Gas Analyzer) equipped with two channels comprising a 10 m Rt-Molsieve 5A column and an 8 m Rt-Q-Bond column, respectively, and thermal conductivity detectors (TCDs). On the other hand, the liquid samples were characterized by using a high-performance liquid chromatograph (Shimadzu, HPLC), furnished with two detectors (RID-10A and PDA 212 nm) and a Rezex ROA Organic acid 300 × 7.8 mm column; 5 mM H_2_SO_4_ aqueous solution was used as the mobile phase. The volatile compounds were also characterized by using a gas chromatograph (Perkin Elmer GC, Clarus 580) equipped with a head space, a Stabilwax-DA column, and a mass spectrometer detector (MSD, SQ8 S).

### 2.4 Thermocatalytic CO_2_ Reduction Tests

**Figure 2:**
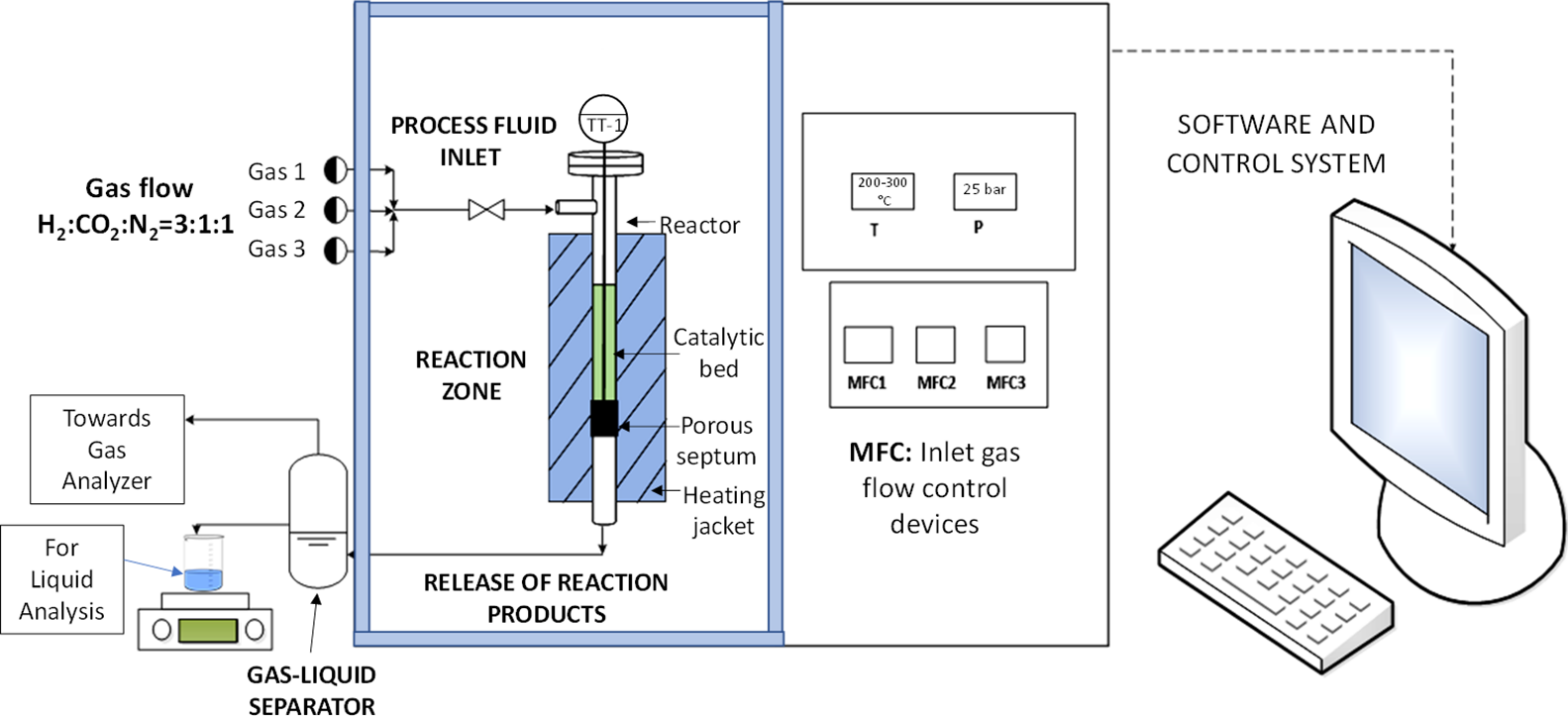
Simplified conceptual scheme of the thermochemical CO_2_ conversion setup.

The catalytic powders were previously pelletized at 100 bar. The pellets were then crushed in a mortar and sieved in a size range between 250 and 500 μm. This size range is required to reduce the pressure drop in the catalytic bed, but without making the mass transfer the controlled phenomenon of the process. Then, the sample (1.5 g of small particles) was tested in a TC test unit using a vertically arranged stainless-steel reactor (i.d. 8 mm), which is positioned in an insulated oven. The sample was previously treated for 3 h in a stream of 10 vol % H_2_/N_2_ (60 *N*L/h) at 2 bar and 350 °C for reducing the Cu_2_O to metallic Cu. Subsequently, a 20 h stability test was performed at constant conditions 25 bar, 270 °C (oven temperature), and 20 *N*L/g/h with a H_2_/CO_2_/N_2_ molar ratio of 3:1:1 to analyze the stability of the catalytic performances. Lastly, each catalyst was tested at 25 bar, 20 *N*L/g/h, and H_2_/CO_2_/N_2_ molar ratio of 3:1:1, ranging the temperature between 200 and 300 °C to evaluate the catalytic activity. The reactor outlet gases were measured online with a gas chromatograph system (7890B of Agilent technologies) by using a TCD and a flame ionization detector (FID). The TC test bench consists of four sections: (i) feeding and regulation of the fluid inlet; (ii) insulation and heating of the tubular reactor; (iii) gas–liquid separation; and (iv) analysis of reaction products. The simplified setup of the CO_2_ hydrogenation process is shown in Figure 2.

## 3 Results and Discussion

### 3.1 Physical–Chemical Characterization of Cu and Cu/ZnO Catalysts

#### 3.1.1 Fresh Powder Catalysts

**Figure 3:**
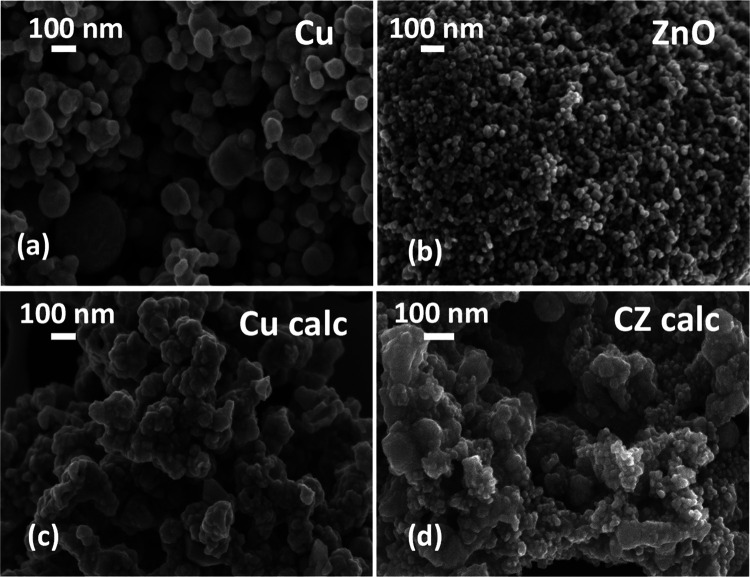
FESEM images of (a) Cu fresh nanopowder; (b) ZnO fresh nanopowder; (c) Cu nanopowder calcined at 150 °C for 2 h, and (d) catalytic mixture of Cu calc and ZnO nanoparticles (CZ calc).

The FESEM micrographs of the fresh catalysts are shown in Figure 3. The as-received commercial copper contains abundant spherical-like particles with a not uniform average size: particles with different sizes from 40 to 200 nm were detected (see Figure 3a). Instead, Figure 3b shows that the zinc oxide particles have almost the same dimension of about 25 nm. Figure 3c shows the increased grain size of the copper particles owing to the calcination process. It is ascribed to several neighbor particles fused by melting their surfaces, increasing the particle size due to the coalescence/sintering mechanism. For this reason, the hand-made catalytic mixture (CZ calc) presents a nonuniform distribution of shapes and strong agglomeration, which may be the result of a naturally occurring interaction between the Cu and Zn nanoparticles, as shown in Figure 3d.

**Figure 4:**
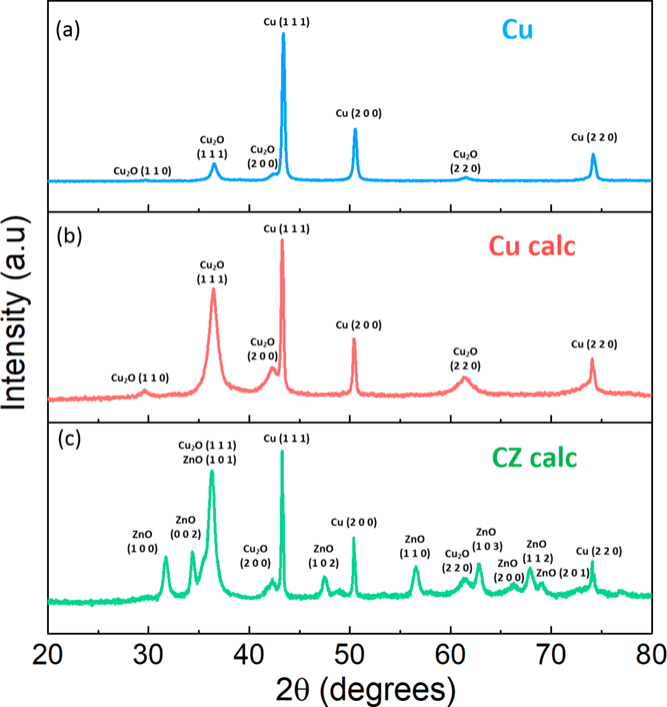
XRD patterns of (a) Cu fresh nanopowder; (b) Cu nanopowder calcined at 150 °C for 2 h; and (c) catalytic mixture of Cu calc and ZnO nanoparticles (CZ calc).

The XRD patterns of catalytic mixtures are compared with the pure copper powder in Figure 4 to understand the present crystalline phases. As can be seen from Figure 4a, the defined reflections of commercial copper nanoparticles cannot be assigned to only the metallic Cu crystalline phase (JCPDS number: 01-089-2838) because Cu_2_O (Cuprite, JCPDS number: 01-077-0199) diffraction peaks were also detected. It is well known in the literature that Cu^1+^ or a mixture between Cu^1+^ and Cu^0^ showed high C_2_ products yield during CO_2_ electroreduction in aqueous solutions.^42−44^ Hence, the commercial Cu powder was calcined at 150 °C for 2 h to increase the Cu^1+^ crystallites. The Cu^1+^/Cu^0^ peak ratio increased after the calcination treatment, while Cu^2+^ was not identified in the XRD patterns, as shown in Figure 4b. Figure 4c shows the crystalline structure of the CZ catalytic mixture. It presents the peaks related to the hexagonal wurtzite crystalline phase of ZnO (JCPDS number: 01-089-7102) and the same mixture of Cu^1+^/Cu^0^ present in the Cu calc sample. It can be seen that more intense and broader Cu^1+^ diffraction peaks were detected after calcination, indicating that an increased amount of small Cu_2_O crystallites was formed when the particles were subjected to the 150 °C treatment. The crystallite size of each phase was calculated from the Debye–Scherrer equation (see Table 1).

**Table 1:** Main Textural Parameters of the Catalytic Mixtures

Catalyst	BET surface area, m^2^ g^–1^	Total pore volume, cm^3^ g^–1^	Crystallite size, nm		
			(111) facet of Cu^0^	(111) facet of Cu^1+^	(100) facet of ZnO
Cu	4	0.010	32	13	
Cu calc	6	0.015	31	8	
CZ calc	16	0.065	31	10	15

From the morphological analysis of the powders, it could be observed that the CZ calc sample has similar characteristics with respect to the Cu calc, although it contains 35 mol % of ZnO nanoparticles. Incorporating ZnO into the Cu-oxide-derived particles increased the nitrogen uptake, indicating a wide pore size distribution, as shown in Table 1. The porosity and particle size of the catalyst can influence mass transport, adsorption/desorption of intermediates in the catalytic layer, and, consequently, the obtained product distribution.

#### 3.1.2 Fresh and Tested Electrodes

As mentioned above, the catalyst particles were mixed with the MWCNT, a solution of Nafion and isopropanol (see Section 2.1) to be deposited on the surface of the working electrode (porous carbon paper), forming the catalytic layer that acts as the cathode. In this regard, the Cu calc and CZ calc electrodes were characterized before and after the tests to study the well-known phenomena of electrocatalyst reconstruction and its influence on the here-observed product distribution.

**Figure 5:**
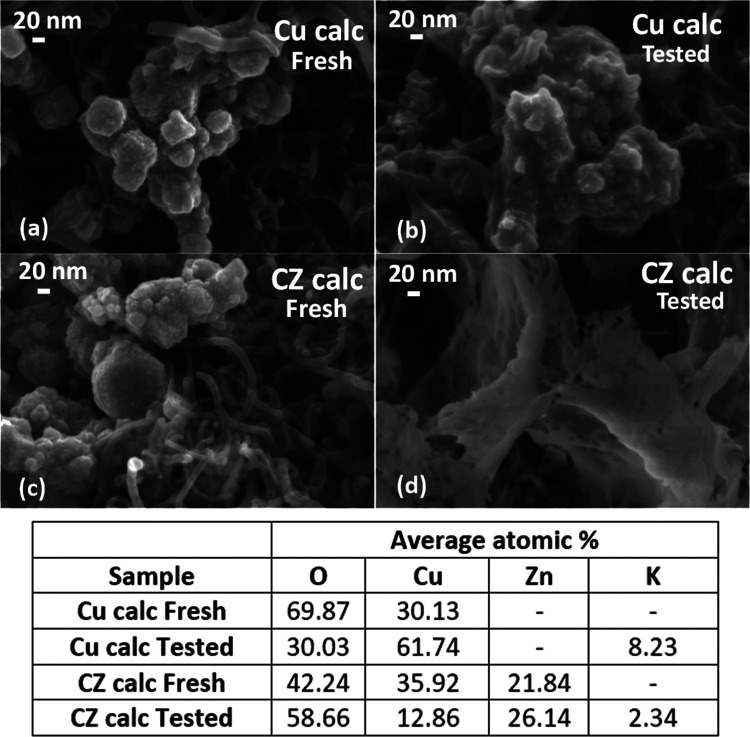
FESEM images of electrodes of 1.5 mg cm^–2^: (a) Cu calc fresh; (b) Cu calc tested; (c) CZ calc fresh, and (d) CZ calc tested. In the case of tested electrodes, the EC CO_2_ R was carried out at a constant potential of −1.4 V vs RHE for 2 h. The table contains the compositions of the elements, as obtained from EDS analyses on each electrode.

Figure 5 shows the FESEM micrographs of the corresponding Cu calc and CZ electrodes. It is evident that the morphology of these electrodes was modified after 120 min of EC CO_2_R at a constant potential of −1.4 V vs RHE (see Figure 5b,d). The micrograph of the tested Cu calc electrode (Figure 5b) evidences sintered and more agglomerated particles than in the fresh electrode (Figure 5a). The EDS analysis demonstrated that those particles are Cu-enriched because the Cu/O atomic ratio passed from 0.4 in the fresh electrode to 2.0 in the tested one. Correspondingly, the XRD bulk analyses of the catalytic layer show an increase from 12 to 50% of the metallic copper amount and a decrease in the crystalline Cu_2_O from 78 to 33% (see Table S1 in the Supporting Information). The morphological changes are more evident in the case of the CZ calc electrodes than in the Cu calc one. The presence of ZnO with the copper nanoparticles promoted the full catalyst restructuration with flake formation after the co-electrolysis of CO_2_ at −1.4 V vs RHE for 2 h, as shown in Figure 5d. As demonstrated by EDS (Figure 5) and XRD (Table S1, Supporting Information) analyses, those flakes are constituted by a Zn-enriched amorphous structure containing metallic copper in the bulk. In contrast to the Cu calc sample, in this case, the Cu/O ratio in the bulk of the catalytic layer decreased from 0.9 to 0.2, while the Zn/Cu ratio increased from 0.6 to 2.0.

**Figure 6:**
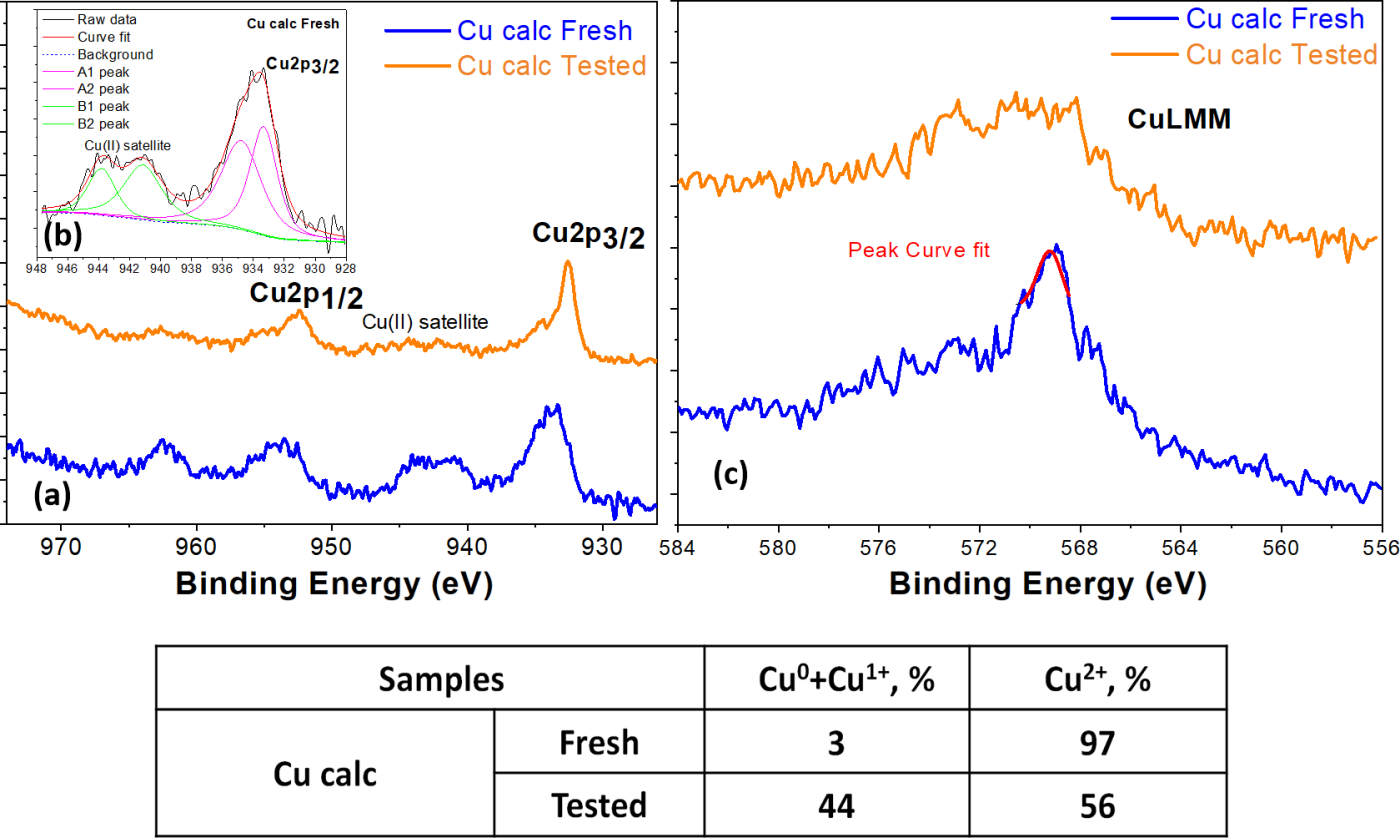
XPS high-resolution spectra for Cu2p doublets (a), the deconvolution peaks of the Cu2p spectra for Cu calc fresh in the inset (b), and Auger CuLMM region (c) for Cu calc fresh and tested electrodes. In the table, the percentage of oxidation states of copper calculated from the Cu2p_3/2_ peak deconvolution procedure^45^ on the surface of the Cu calc fresh and tested electrodes was reported. In the case of the tested electrode, the EC CO_2_R was carried out at a constant potential of −1.4 V vs RHE for 2 h.

On the other hand, the Cu2p doublet region of the Cu calc electrodes acquired by XPS in HR mode is shown in Figure 6a. At the surface, the fresh electrode displays a typical spectrum related to only Cu^2+^, while the tested sample shows a typical structure related to the mixed oxidation states of copper (Cu^0^, Cu^1+^, and Cu^2+^).^45^ It is worth noting that the calcination treatment of the pristine powder was performed at a low temperature (150 °C); therefore, only superficial passivation could be verified. On the other hand, as mentioned in Section 2.1, the deposition of the catalytic ink (containing the Cu calc) was carried out by placing the carbon paper on a heating plate at 120 °C, and after deposition, it was kept on it for 15 min to ensure complete solvent evaporation. Thus, it is also hypothesized that the surface of the electrode was further oxidized during its preparation. For this reason, it presents a high amount of superficial Cu^2+^. The Cu2p peak is complicated to be deconvoluted because of both the presence and overlapping of several satellites and shake-up peaks for each oxidation state. In order to obtain more details, the Auger CuLMM region was also obtained (see Figure 6b).

The resulting modified Auger parameter is approximately 1851 eV for the Cu calc fresh sample, which corresponds to the average oxidation state (AOS) of Cu^2+^, indicating that its surface is mainly composed of CuO, with a thickness of at least 5–10 nm (the sensible depth for XPS). Results in the table in Figure 6 further confirm the high percentage of Cu^2+^ on the surface of the Cu calc fresh electrode, which was estimated through the method developed by Biesinger et al.^45^ The formulas used to calculate the relative amount of Cu species are listed in Section S5 of the Supporting Information. Indeed, by fitting the Cu2p_3/2_ peak and its related satellite, it is possible to evaluate the percentage of Cu^2+^ and Cu^0^ + Cu^1+^ with respect to all the present copper species. In contrast, XRD results revealed the coexistence of metallic Cu^0^ and Cu^1+^ in the bulk of the Cu calc powder sample, while Cu^2+^ was absent, as shown in Figure 4b. It could be explained with the temperatures of Hüttig [0.3 T of melting (Kelvin)] and Tammann [0.5 T of melting (Kelvin)].^46^ Practically, at the Hüttig temperature, the surface atoms begin to move, while at the Tammann temperature, the bulk atoms also move. In particular, for metallic Cu, these two temperatures are 134 and 405 °C. Therefore, because calcination took place at 150 °C (<Tammann T), it is realistic to think that only the surface atoms react with oxygen forming Cu^2+^, while deeper in bulk, they cannot react because the diffusion of the O atoms is too slow. Therefore, in bulk, there are both Cu^1+^ and Cu^0^, as shown by the EDS (table in Figure 5) and XRD data (Table S1) discussed earlier. Regarding the tested electrode, the modified Auger parameter is about 1849 eV, typical of Cu^1+^. Indeed, the percentage of Cu^0^ + Cu^1+^ increased, indicating that the electrode surface was reduced after the co-electrolysis of CO_2_ and, therefore, the surface Cu^2+^ abundance decreased in the tested sample.

**Figure 7:**
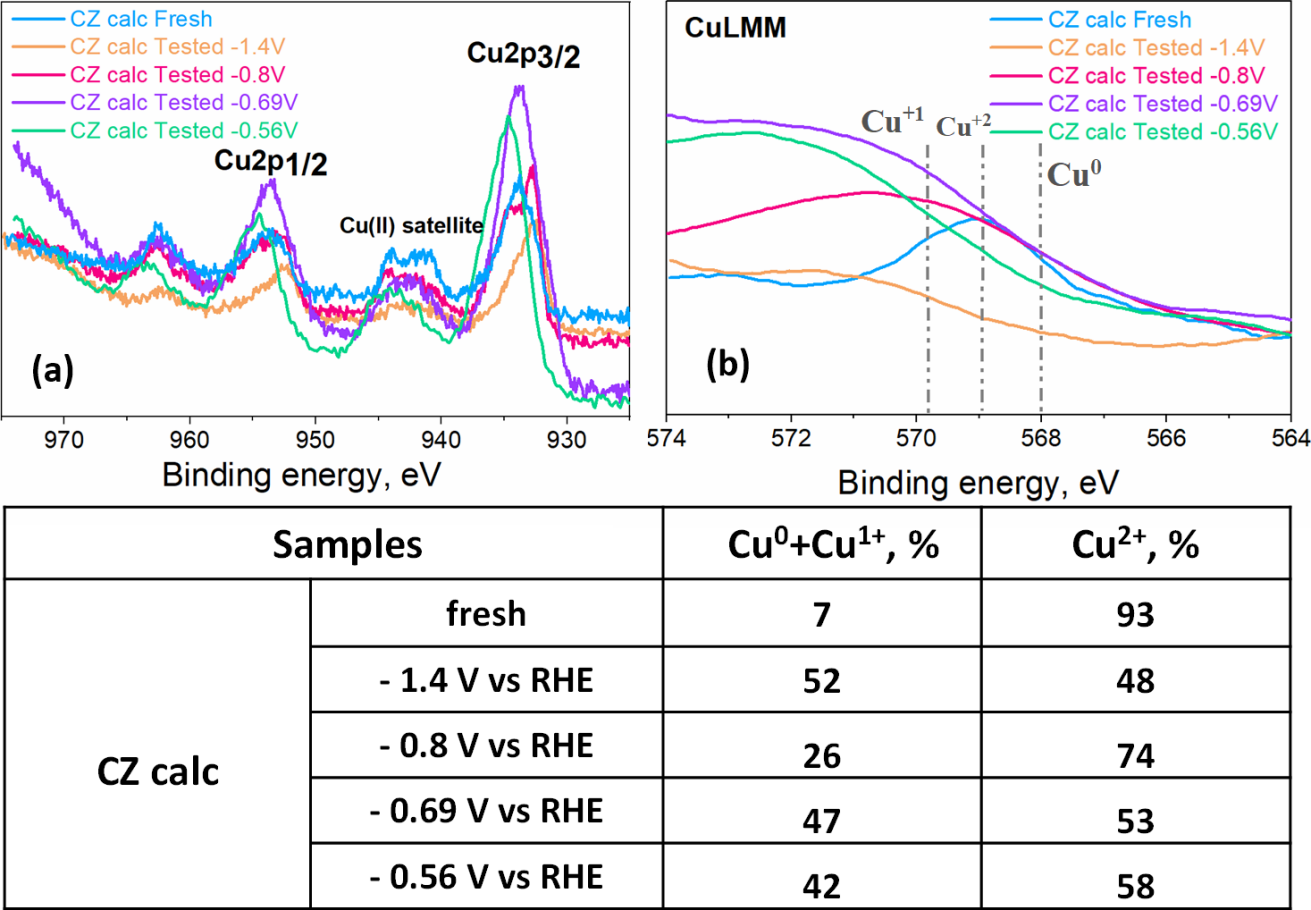
XPS high-resolution spectra for Cu2p doublets (a) and Auger LMM region (b) of CZ calc fresh and tested electrodes at different potentials. In the table, the percentage of oxidation states of copper calculated from Cu2p_3/2_ peak deconvolution procedure^45^ on the surface of the CZ calc fresh and tested (at −1.4, −0.8, −0.69, and – 0.56 V vs RHE) electrodes were reported.

XPS measurements were also performed on the CZ electrodes to investigate the chemical composition of their surface. As mentioned before, the Auger signature is more sensitive to changes in the Cu oxidation state than the Cu 2p_3/2_ core-level signature. Indeed, in Figure 7a, the corresponding Cu2p spectra show some small peak shifts between the tested samples at different potentials because of the mixed oxidation states of copper. Instead, the fresh CZ calc electrode exhibits features associated with the presence of Cu^2+^ on the surface, which could also be observed in the corresponding Cu LMM spectrum, as shown in Figure 7b. The XRD analysis of this powder catalyst (Figure 4c) is similar to that of the Cu calc catalyst but with the presence of the hexagonal wurtzite crystalline phase of ZnO because this mixture was prepared by hand-mixing without any aggressive treatment. Therefore, it has the coexistence of metallic Cu^0^ and Cu^1+^ in bulk.

Regarding the Cu LMM spectrum, more changes were observed. As shown in Figure 7b, the broad and asymmetrical Cu LMM spectra in the case of tested electrodes demonstrate the presence of more components. The binding energies of the main Auger peaks are measured at 568.0, 569.8, and 568.9 eV for Cu^0^, Cu^1+^, and Cu^2+^, respectively.^47^ In this regard, the Cu LMM of the electrodes subjected to the lowest applied potentials (−0.8, −0.69, and – 0.56 V) shows a mix of the three Cu oxidation states, while the electrode tested at the highest applied potential presents a structure mostly associated with Cu^1+^. Therefore, it is hypothesized that the percentage of the mix Cu^0^ + Cu^1+^, estimated through the method developed by Biesinger^45^ and shown in the table of Figure 7, is due mainly to Cu^1+^ rather than the Cu^0^ oxidation state. It is proved by the absence of the Cu^0^ shoulder at 565–564 eV in the CuLMM spectra in all the tested sample graphs. The prevalence of superficial Cu^+1^ rather than Cu^0^ species in the CZ tested samples could be ascribed to the stabilizing role of the ZnO matrix toward this copper oxide and the high degree of catalyst restructuration that occurred in the presence of zincite, as shown in Figure 5. Indeed, the surface elemental composition calculated from the survey XPS spectra (Table S6) revealed an enrichment by Cu, Zn, and O of the CZ electrode surface after testing (see Section S5 in the Supporting Information), with a 2.6-fold increase in the Zn/Cu ratio with respect to the fresh sample and a consequent covering of the MWCNTs used to increase the conductivity of the catalytic layer.

**Figure 8:**
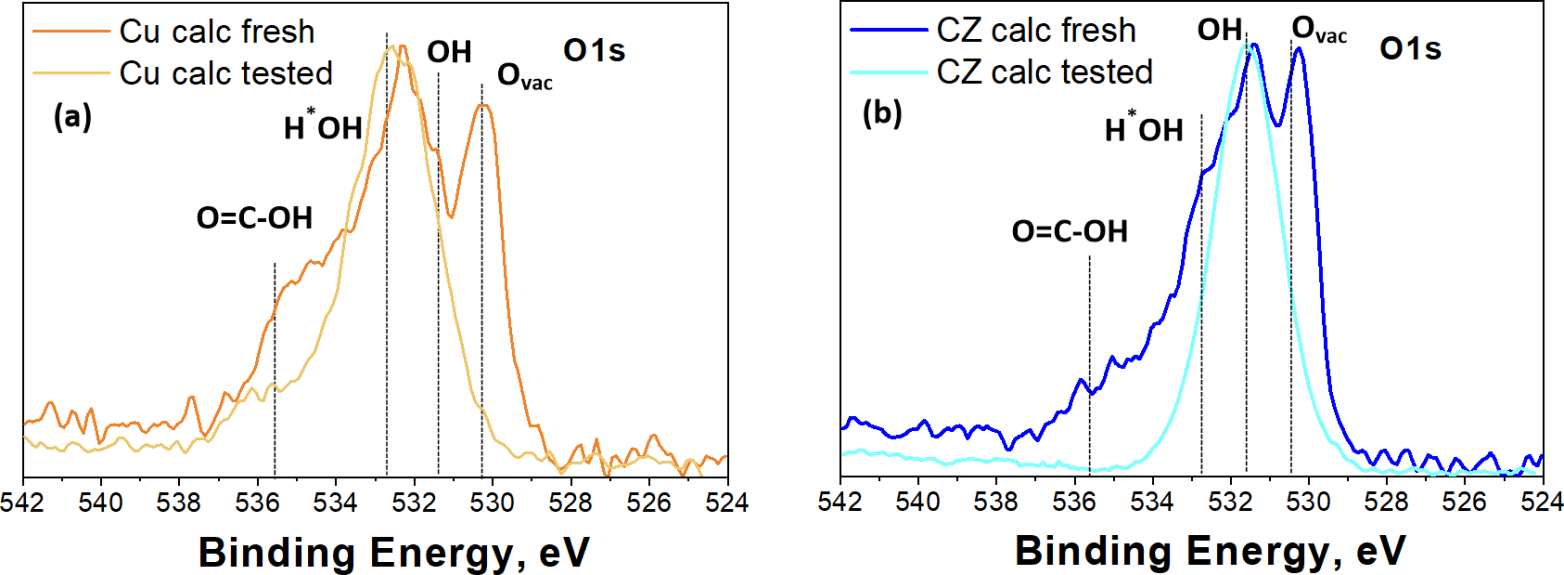
High-resolution O 1s XPS spectra of the prepared electrodes: Cu calc fresh and Cu calc tested (a) and CZ calc fresh and CZ calc tested (b). In the case of tested electrodes, the EC CO_2_R was carried out at a constant potential of −1.4 V vs RHE for 2 h.

XPS results also reveal the existence of abundant oxygen vacancies in both fresh electrodes (see Figure 8), which could increase the binding affinities to the key intermediates that favor the EC CO_2_ conversion to more reduced and useful products. In addition, both fresh and tested electrodes presented OH and H*OH species on their surface, which demonstrate the pertaining basicity on the samples even after restructuration. The existence of abundant oxygen vacancies and basic sites should promote the CO_2_ adsorption and its conversion.^48^

### 3.2 Electrochemical Measurements

#### 3.2.1 Electrochemical Behavior in the Working Electrolyte

**Figure 9:**
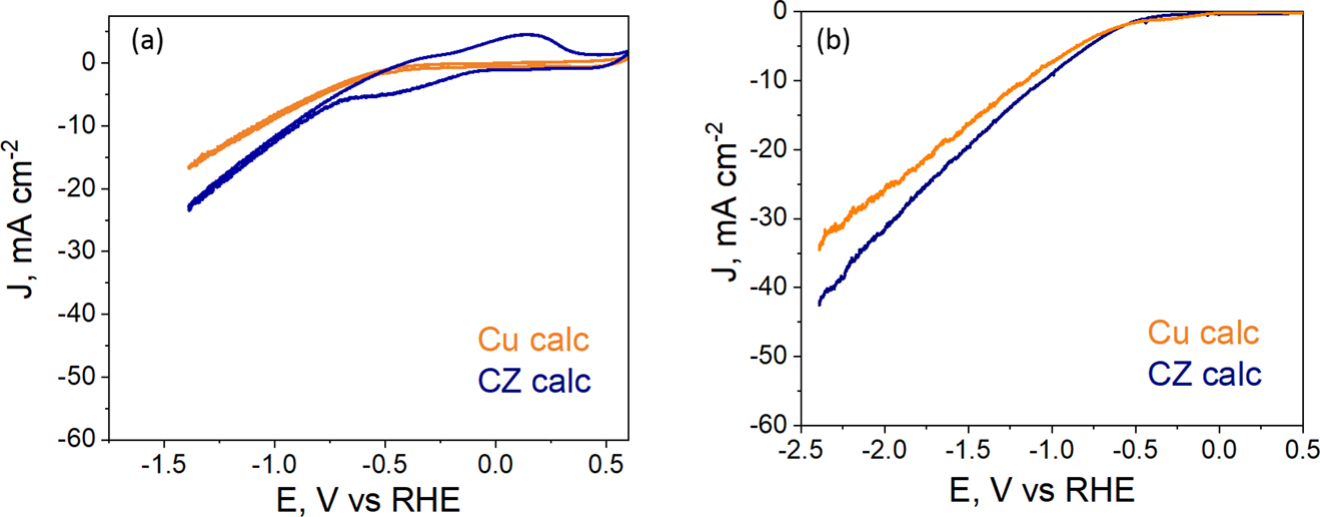
CV responses (a) linear polarization curves (b) obtained for Cu calc and CZ calc electrodes in a CO_2_-saturated 0.1 M KHCO_3_ aqueous solution.

Initially, the system was bubbled with N_2_ for 20 min at a flow rate of 10 mL min^–1^ in order to degas the working electrolyte. Then, blank CV was performed on the N_2_-purged electrolyte by scanning the electrode in a potential window between 0.5 and −1.4 V vs RHE. The same procedure was employed in the CO_2_-saturated working electrolyte after bubbling CO_2_ on it for 30 min with a flow rate of 10 mL min^–1^. The electrochemical measurements were carried out by continuously bubbling the gas into the electrolyte. Figure 9a shows the reduction/oxidation features of the catalysts in the CO_2_-saturated solution. It is worth noting that the CZ calc catalyst appears to be more active because there is a lower onset potential (at approximately −0.2 V vs RHE) in CO_2_ flow, and its EC activity (the absolute current density) is higher than that of the bare Cu calc. In addition, the CV of CZ calc demonstrates two redox peaks (see Figure 9a). The anodic–cathodic branches could be associated with the oxidation (positive current) or reduction (negative current) of intermediates adsorbed on the catalyst surface. It is important to mention that ZnO is a catalyst with more CO-selective sites than the bare Cu.^37^ For this reason, this behavior could be associated with the capture of CO molecules from the reduction of CO_2_, as indicated by the striping voltammetry of CO oxidation. It can also be seen that CZ mixture catalysts have a capacitive behavior. It is probably due to the formation of a double electric layer established between the surface of the CZ catalyst and the electrolyte solution near the electrode. It could be attributed to the presence of mixed metal oxides that are less conductive.

LSV measurements were carried out on different Cu calc and CZ calc electrodes with the same catalyst loading and under the same operating conditions to demonstrate that the EC activity of the here-studied materials is reproducible. The curves confirming the similar behavior of all the repeated tests are shown in Section S3 in the Supporting Information. The linear polarization curves in Figure 9b show an increase in the final total current density (the total activity of the electrode) of approximately 24% with the CZ calc electrode at −2.4 V vs RHE, which seems to correlate with the role of the metal oxides in enhancing CO_2_ adsorption and conversion. These results agree with the XPS measurements shown in Figure 8, that is, the existence of abundant oxygen vacancies on the catalyst surface promotes the adsorption of CO_2_ and its reaction intermediates.^49^

#### 3.2.2 Electrochemical Activity toward the CO_2_ Reduction Reaction

**Figure 10:**
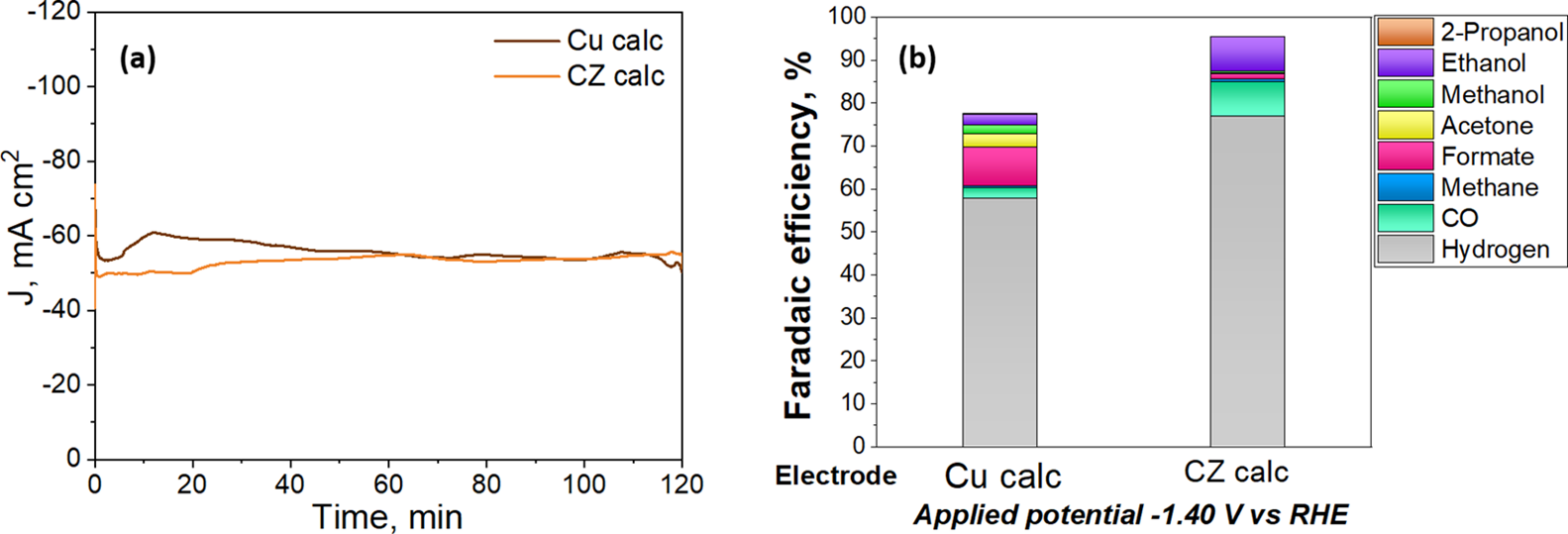
(a) Evolution time of the current density and (b) FE for different products formed after 120 min of EC CO_2_R at a constant potential of −1.4 V vs RHE for Cu calc and CZ calc electrocatalysts.

The influence of the Cu-based electrodes for the EC CO_2_R was studied through a CA at −1.4 V vs RHE for 120 min under CO_2_-saturated KHCO_3_ solution. From Figure 10a, it is possible to see that a high cathodic current density was obtained when these calcined catalysts were used as working electrodes. In the case of the CZ calc, no significant current density changes were observed after 120 min of CO_2_ coelectrolysis. In contrast, the generated current density response of the Cu calc presents an increase up to 3% during the first 20 min, and it reaches the same current density of the CZ catalyst (approximately −53 mA cm^–2^) after 60 min. This behavior can be attributed to the reduction of the catalyst during the experiment until its stabilization.

The total product distribution and Faradaic efficiencies obtained with the Cu calc and CZ calc electrocatalysts are given in Figure 10b, Table S2, and Table S3 (Supporting Information). Clearly, the Cu calc catalyst evidenced a remarkably higher selectivity to C_1_ products (FE_formate_ + FE_CO_) than the CZ material. Instead, the CZ catalyst showed higher ethanol (C_2_) selectivity than the bare copper material, reaching a FE_EtOH_ of approximately 8%. From the XPS measurements, a lower percentage of Cu^0^ + Cu^1+^ was present in the surface of the Cu calc tested electrode than in the CZ calc tested one, as shown in the tables in Figures 6 and 7. Thus, the Cu^0^ + Cu^1+^ percentage on the surface of these electrodes is directly proportional to the reached Faradaic efficiency toward ethanol. On the other hand, XRD (see Section S2, Supporting Information) and EDS analyses (Figure 5) confirm copper reduction also in the catalyst bulk in both electrodes, when the negative potential was applied under the CO_2_ flow: the Cu_2_O originally present in the Cu calc fresh electrode was partially reduced to Cu^0^; instead, there is not any trace of copper oxides in the CZ calc tested sample, which was entirely reduced in the bulk under reaction conditions. Recent literature revealed the possibility of inducing C–C coupling and promoting the formation of C_2+_ products if Cu^1+^/Cu^0^ interfaces are stabilized.^50,51^ The here-reported results further confirm that the presence of the reduced species of copper (Cu^1+^ and Cu^0^) at the catalyst surface are the main active sites for the CO_2_ reduction reaction to C_2+_ alcohols. In addition, it is evident that the presence of ZnO in the CZ calc sample has also a role in Cu^+1^ stabilization during the catalyst restructuration and the improved ethanol production. Herein, the selectivity toward more reduced products (i.e. ethanol) appears to correlate with CO formation. It seems that the catalyst should be active enough for producing CO but should also have suitable binding energy toward the formation of *CO intermediate for producing C_2+_ products. Indeed, ZnO is a CO-generation catalyst. Therefore, the CO productivity reached by the CZ calc electrode was twofold higher than that of the Cu calc electrode, as well as its conversion was 15% higher than the latter, under the same reaction conditions (see Tables S2 and S3 in the Supporting Information). Hence, the enriched ZnO surface increases the local CO concentration, allowing a higher formation of the key CO-adsorbed intermediate (*CO) at the Cu^+1^/Cu^0^ interface that, subsequently, is transformed by dimerization reactions (namely, *CO–*CO or *CHx–*CO) into C_2+_ products like ethanol.^42,52,53^ These findings agree with a recent work on ZnO@Cu-derived and Cu@ZnO-derived catalysts that showed selectivity for ethanol and methane, respectively. Experimental results and DFT simulations show that a higher Zn content increases the local CO concentration and enables a tandem conversion mechanism, determining the selectivity shift from CH_4_ to ethanol.^52^ Similarly, it was found an enhanced ethanol selectivity at the terraces of a Cu–Ag bimetallic system, via a *CHx–*CO coupling pathway, because of the CO-enriched environment generated by Ag nanospheres.^53^

**Figure 11:**
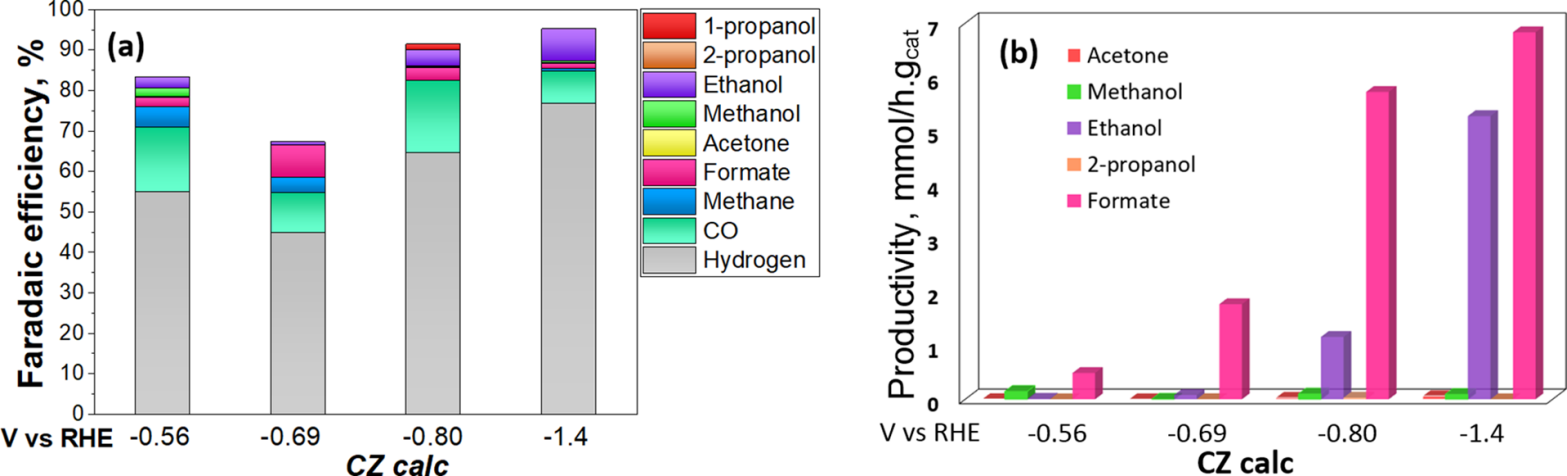
(a) FE for different products formed after 120 min of EC CO_2_R at different applied constant potential (−0.56, −0.69, −0.80, and −1.4 V vs RHE) and (b) productivity of the main liquid products at the different working potentials of the CZ calc electrocatalyst.

Additionally, to study the influence of the applied potential on the CO_2_R products, CA measurements under the CO_2_-saturated electrolyte were performed at other three lower potentials (−1.14, −0.80, and – 0.69 V vs RHE) for 120 min, under the same reaction media and using the CZ calc material as the electrocatalyst. The whole product distribution is listed in Table S4, while the Faradaic efficiency performances are shown in Table S5 (Supporting Information). From Table S4, it is possible to appreciate that the CO_2_ conversion increased as the negative applied potential increased. However, the best CO_2_ conversion reported here is still not high enough for an industrial application. We have recently demonstrated through simulations that, to render electrocatalysis a promising route to reduce CO_2_ to value products, the EC technology has to be scaled up considering recycling the unreacted CO_2_ gas to increase the overall carbon dioxide conversion and productivity.^3^ Nevertheless, further research is needed to optimize catalyst performance (achieving FE > 90%) and cell designs to reduce mass-transfer limitations and reach >100 mA cm^–2^. Figure 11a shows an increase in the reaction kinetics of the CO_2_ reduction reaction (CO_2_RR) toward C_2+_ (ethanol) product as the negative applied potential was increased from −0.69 to −1.4 V vs RHE. It should be pointed out that 1-propanol was detected as a product at −0.80 V vs RHE with a FE_1PrOH_ of approximately 2%. The maximum CO Faradaic efficiency (approximately 18%) was also achieved at that applied potential, confirming the previously explained link between CO production and C_2+_ product generation.

**Figure 12:**
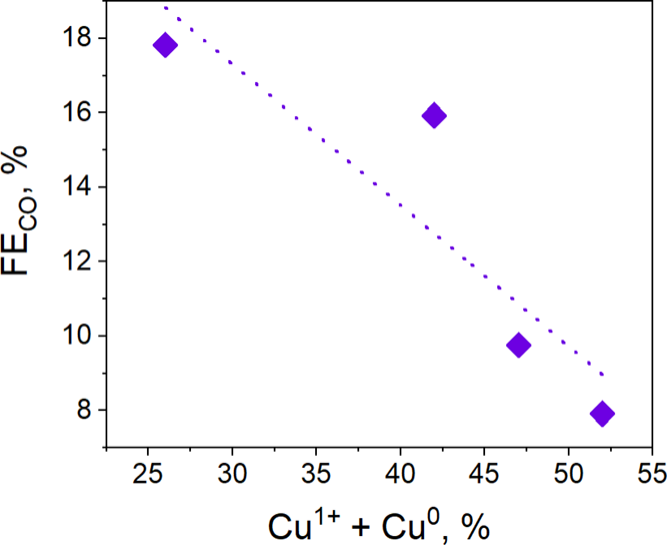
Relationship between FE_CO_ and Cu^0^ + Cu^1+^ percentage of the CZ calc tested electrode for the EC CO_2_R at different applied constant potentials (−0.56, −0.69, −0.80, and −1.4 V vs RHE). See also data in the table of Figure 7.

Figure 11b shows that the productivity (mmol/h g_cat_) of formate and ethanol increased by increasing the negative applied potential, reaching ∼6.85 and ∼5.27 mmol·g_cat_^–1^·h^–1^, respectively, at −1.4 V vs RHE. From the XPS measurements shown in Figure 7, the electrodes tested at different potentials evidence a mix of Cu^0^, Cu^1+^, and Cu^2+^ oxidation states. Consequently, it seems that by increasing the applied energy, the barrier of the *CO dimerization is reduced, inducing a high activity for the EC CO_2_ reduction toward C_2+_ products. Once again, the amount of Cu^0^ + Cu^1+^ on the electrodes after testing appears to correlate with ethanol formation. Figure 12 shows that the higher is the Cu^0^ + Cu^1+^ percentage, the lower is the CO Faradaic efficiency of the reaction. It seems that as the Cu^0^ and Cu^1+^ species increases, *CO intermediate stabilization is enhanced, and thus, CO is not easily desorbed as a gaseous product. It is ascribed to the fact that the Cu^1+^/Cu^0^ interface enhances the *CO binding energy^54^ and promotes the *CO intermediate dimerization toward C_2+_ products.^50,51^ Indeed, the electrode that presented the highest Cu^0^ + Cu^1+^ percentage (52%) achieved the highest FE toward ethanol (approximately 8%). On the other hand, the Cu species stabilized at −0.8 V, having the lowest Cu^1+^ + Cu^0^ amount, and a higher Cu^+2^ was the most suitable to produce C_3_ alcohol like 1-propanol.^51^

### 3.3 Thermochemical Activity toward the CO_2_ Reduction Reaction

The CZ calc catalyst was tested in a TC test unit to compare its performance concerning the products obtained using the same catalyst under CO_2_ electrochemical conditions and to literature data on heterogeneous catalysts for methanol synthesis. The research activity confirmed the synergy between Cu and ZnO particles for MeOH synthesis from CO_2_ and H_2_. Indeed, the metal–metal oxide (i.e. Cu–ZnO) contact is responsible for the increase in methanol productivity on these types of catalysts. In more detail, ZnO increases the basicity of the surface, favoring the CO_2_ adsorption capacity of a Cu–ZnO catalyst directly. In addition, the intimate contact between Cu and ZnO phases allows Zn atoms to migrate, forming a Cu–Zn alloy on the surface of Cu particles and O vacancies in the structure of ZnO particles.^55^ Lastly, Le Valant et al. have mathematically correlated the catalytic activity in methanol synthesis with the concentration of contact points (by assuming a spherical geometry of the particles) between the two phases.^55^ In conclusion, greater interaction between Cu and ZnO favors a higher MeOH productivity because of the enhanced H_2_ dissociation and adsorption capacity, and the more intimate contact between the two phases and the formation of O vacancies.^55^ More in detail, Table 2 shows the variations of the textural properties of the CZ calc catalyst after the TC tests. The specific surface area does not change significantly, while the total pore volume decreases by about one-third, which is probably due to a rearrangement of the structure of the catalytic particles under the reaction conditions.

**Table 2:** Comparison of the Textural Properties of the Calcined and the Aged CZ Calc Catalyst

Catalyst	BET surface area, m^2^ g^–1^	Total pore volume, cm^3^ g^–1^	Crystallite size, nm		
			Cu	Cu_2_O	ZnO
fresh CZ calc	16	0.065	31	8	15
aged CZ calc	18	0.041	61		25

On the other hand, as shown in Supplementary Figure S2, Cu^1+^ in the cuprite (Cu_2_O) was completely reduced to metallic Cu^0^ during the TC tests, and, as expected, the crystallites sintered together by forming larger crystallites, as shown in Table 2. In fact, the Cu^0^ crystallite size doubles from 31 to 61 nm, while the ZnO crystallite size increases from 15 to about 25 nm. The semiquantitative analysis^56^ of the ex situ X-ray diffractogram of the aged CZ calc catalyst revealed that the composition is approximatively 75 wt % Cu and 25 wt % ZnO, which is consistent with the expected results.

**Figure 13:**
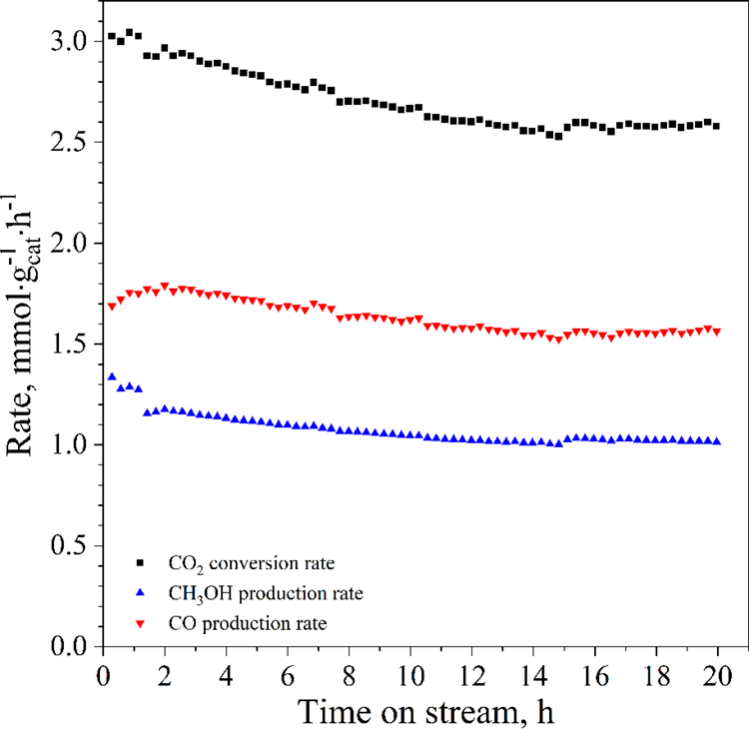
CO_2_ conversion rate, methanol, and CO space–time yields during the 20 h stability test on CZ calc (reaction conditions: 25 bar, 270 °C, 20 *N*L·g_cat_^–1^·h^–1^ and H_2_/CO_2_/N_2_ molar ratio equal to 3/1/1).

Concerning the TC performance, Figure 13 illustrates the methanol productivity during the 20 h stability test. What stands out from these experiments is that the CZ calc catalyst exhibited an initial CO_2_ conversion of ∼1.73% that decreases during the test, reaching ∼1.43% at the end of the 20 h stability test. Similarly, methanol productivity diminishes by ∼27% from ∼1.4 to ∼1.02 mmol·g_cat_^–1^·h^–1^, while the CO productivity decreases from ∼1.75 to ∼1.55 mmol·g_cat_^–1^·h^–1^. Notwithstanding, both methanol and CO selectivities remained constant at 40 and 60%, respectively, during the test. It means that catalytic deactivation affected the reaction rate, reducing the number of active sites, but it did not affect their nature, and accordingly, the reaction mechanism. Le Valant et al. (2015) have demonstrated that an increase in the particle size reduces the number of contacts between Cu and ZnO, which are the active sites responsible for the enhanced activity in the methanol synthesis of these bimetallic catalysts.^55^ These performances are consistent with those reported in the literature for Cu/ZnO binary catalysts used in CO_2_ hydrogenation to methanol under similar reaction conditions.^57^ More in detail, pure copper-based catalysts exhibited an extremely low activity in CO_2_ hydrogenation, while the presence of ZnO enhances the activity of the binary catalyst.^57^ It means that the presence of both Cu and ZnO in the CZ calc catalyst improves the performance of the catalyst in methanol synthesis because both two phases are active in CO_2_ hydrogenation to methanol.^57,58^ However, the performance of the CZ calc catalyst did not achieve those of commercial catalysts for methanol synthesis as this physical mixture does not allow for obtaining an equally effective Cu/ZnO composite catalyst.^3^ As illustrated in Figure 13, the major concern of this reaction at high temperatures and pressures is related to catalyst deactivation. It was ascribed to two simultaneous effects: (i) the production of water, which can oxidize metallic copper to metal oxides during testing;^59^ (ii) the sintering of metallic particles, which reduces the exposed active surface area and, therefore, the number of contact sites between the two phases (i.e., Cu and ZnO) is reduced.

During the stability test, the CZ calc showed low activity in the TC CO_2_ hydrogenation route, but it exhibited an enhanced selectivity toward methanol. In contrast, Table 3 summarizes the activity performances of the CZ calc catalyst at the variation of the operative temperature in terms of CO_2_ conversion (ζ_CO_2__), selectivity (S), productivity (PR), and yield (Y) values. Its CO_2_ conversion increases as the temperature increases, and the CZ calc exhibited a higher activity because of a higher production of CO via the endothermic reverse water gas shift (RWGS) reaction (eq 1). At the same time, the thermodynamic equilibrium tends to limit methanol formation from either CO_2_ or CO because of its exothermicity (eqs 2 and 3, respectively).
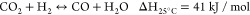
1
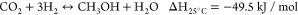
2
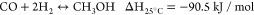
3

**Table 3:** Catalytic Performances of the CZ Calc Catalyst and Thermodynamic Equilibrium (Reaction Conditions: 25 Bar, 20 *N*L·g_cat_^–1^·H^–1^ and H_2_/CO_2_/N_2_ Molar Ratio Equal to 3/1/1)

	CZ calc catalyst					Thermodynamic equilibrium				
*T*	ζ_CO_2__	*S*_CO_	*S*_CH_3_OH_	*PR*_CH_3_OH_	*Y*_CH_3_OH_	ζ_eq, CO_2__	*S*_eq, CO_	*S*_eq, CH_3_OH_	*PR*_eq, CH_3_OH_	*Y*_eq, CH_3_OH_
°C	%	%	%	mmol·g_cat_^–1^·h^–1^	mmol·g_cat_^–1^·g_CO2,INLET_^–1^	%	%	%	mmol·g_cat_^–1^·h^–1^	mmol·g_cat_^–1^·g_CO2,INLET_^–1^
200	0.12	0	100	0.208	0.026	20.88	19.29	80.71	29.23	3.723
225	0.23	0	100	0.399	0.051	18.56	44.04	55.96	18.02	2.295
250	0.62	36.66	63.34	0.681	0.087	18.71	70.47	29.53	9.58	1.220
275	1.46	58.67	41.33	1.047	0.133	20.57	87.06	12.94	4.62	0.588
300	3.41	76.75	23.25	1.375	0.175	23.35	94.65	5.35	2.18	0.278

The catalytic performance of the here-prepared Cu/ZnO catalyst is lower than the most performing catalysts studied for CO_2_ hydrogenation for methanol production.^3^ The best CO_2_ conversion reached with a Cu/ZnO catalyst is higher than 5%, reaching 50% of methanol selectivity. It could be generally accepted that hydrocarbons and multicarbon oxygenates are promoted on Cu nanoparticles higher than 15 nm, whereas CO and H_2_ are favored on smaller ones.^6,44,60−63^ Thus, the higher selectivity toward methanol at low temperatures could be justified by the average particle size of Cu (>40 nm) in the CZ calc catalyst, which promotes methanol synthesis. As mentioned before, increasing the intimate contact between Cu and ZnO phases during the preparation will favor methanol production. Therefore, further optimization in the catalyst preparation is required. Possible strategies could be to change the calcination temperature for modifying the crystallite size of the involved phases.

### 3.4 Divergences and Potential Synergies between EC and TC CO_2_ Conversion

The performed tests on the CZ calc catalyst confirmed that, as expected, the physical mixture of Cu NPs and ZnO exhibited a synergistic interaction in hydrogenating CO_2_. According to the literature, its TC performance strictly depends on the textural properties; moreover, the CZ calc catalyst only promotes the formation of CO and methanol, bearing metallic Cu and crystalline ZnO formed in the catalyst under the H_2_ atmosphere at high temperatures. In contrast, the EC system is extremely complex because it depends on many other aspects (like electrode polarization, CO_2_ solubility in the aqueous media, among others). As well, the catalytic layer transforms continuously during the EC reaction even under ambient conditions. Our results demonstrate that the presence of ZnO in the catalyst leads to the formation of mixed copper oxidation states and Cu^1+^/Cu^0^ interfaces, with relative amounts that depend on the applied potential (see Figure 12), embedded into an amorphous zinc oxide-based matrix that is rich in basic sites (e.g., −OH). Therefore, several products could be produced during the EC tests, such as CO, methanol, ethanol, propanol, methane, ketones, formate, and hydrogen (see Figure 11). The mechanisms behind the formation of these different products should be identified to reach a complete understanding of the EC and TC reactions. However, the reported results demonstrate that the formation and stabilization of the CO intermediate at the catalyst surface is the key for producing high-energy-density products in both processes.

The different selectivity of the CZ catalyst under TC and EC conditions could be explained based on the literature data. First, the activation energy for the CO desorption of Cu^0^ surfaces (i.e. between 12 and 16 kcal/mol) is much lower than that of Cu^1+^ surfaces (i.e. between 18.2 and 22.4 kcal/mol).^54^ This can be explained because Cu^+^ cations have an enhanced σ bonding of CO because of its decreased Cu 4s/4p-derived density of states with respect to metallic Cu surfaces. Thus, the binding energy of *CO at the Cu^0^ catalyst surface in the TC process is lower than that in the Cu^1+^ present in the EC one. It leads to preferential CO production in the TC system, which increases as the temperature increases because of a faster CO desorption rate. The thermodynamically favored RWGS endothermic reaction (eq 1) was observed from both experimental and thermodynamic data shown in Table 3. Instead, under ambient EC CO_2_R conditions, gaseous CO is produced in the CZ catalyst, as it is expected for nanosized Cu–ZnO catalysts,^64^ but it is neither the only CO_2_RR product nor the most prevalent one. Thus, the presence of Cu^1+^ might play an important role in increasing the CO intermediate residence time at the electrocatalyst surface, allowing the formation of more reduced products.

Exothermic methanol production from CO_2_ TC hydrogenation is favored at low temperatures, but because of kinetics limitations, it is usually performed at *T* > 200 °C. Two classes of reaction routes have been proposed in the literature:^65^ (i) the formate pathway, where the HCOO* intermediate formation is considered as the rate-determining step; (ii) the RWGS route, suggesting that CO is formed by eq 1 and then converted to methanol (eq 3). However, based on density functional theory (DFT) calculations, Zhao et al.^66^ recently concluded that the direct hydrogenation of formate is not feasible on Cu(111) because of the high activation barriers for some of the elementary steps, in agreement with the experiments by Yang et al.,^67^ who thoroughly studied HCOO hydrogenation on Cu catalysts by simultaneous mass spectroscopy and infrared spectroscopy techniques. They also found an important role of trace amounts of water in the reaction media: CO_2_ hydrogenation to the hydrocarboxyl radical (trans-*COOH) is kinetically more favorable than formate in the presence of H_2_O via a unique hydrogen-transfer mechanism. The trans-*COOH is then converted into hydroxymethylidyne (*COH) via dihydroxycarbene (*COHOH) intermediates, followed by three consecutive hydrogenation steps to form hydroxymethylene (*HCOH), hydroxymethyl (*H_2_COH), and methanol. Their calculations show that CO hydrogenation to methanol may also follow the *COOH route.^66^

On the other hand, methanol productivities are usually very low in aqueous-based EC CO_2_RR conditions, agreeing with the results presented in this work. It can be explained by kinetic and thermodynamic limitations and the prevalence of reaction pathways, leading to the formation of C_2+_ alcohols and other oxygenates. The competing reaction pathways for EC CO_2_RR to alcohols vs CO or formate products have been reported in previous studies.^2^ As recently found for the TC process,^66^ the formation of *COOH through CO_2_ activation and hydrogenation is the first rate-determining-step (RDS) of the EC CO_2_RR, leading to either formate or CO production after two proton-coupled electron-transfer (PCET) reactions. If the *CO binding energy is high enough, successive PCET reactions can lead to more reduced products, such as CH_3_OH or CH_4_, after a total exchange of 6 and 8 electrons (e^–^) and protons (H^+^), respectively. Water plays a fundamental role as an in situ proton source. However, the reported CZ catalyst was more prone to induce C–C coupling, which requires more than 10 PCET processes (i.e., 12 and 18 e^–^/H^+^ for ethanol and propanol generation, respectively). It can be ascribed to the presence and stabilization (by ZnO) of Cu^1+^ in the electrocatalyst surface. Indeed, Goddard et al.^42^ studied Cu metal embedded in an oxidized catalyst matrix by computational efforts. They unveiled that the electrostatic tension between Cu^+^ and Cu^0^ species at adjacent surface sites increases the EC CO_2_RR efficiency by promoting *CO dimerization. Moreover, Zhang et al.^68^ recombined DFT and X-ray absorption spectroscopy (XAS) experiments and found that oxygen in oxygen-derived Cu (OD-Cu) catalysts plays a critical role in strengthening CO adsorption and boosting C–C coupling to C_2_H_4_. They concluded that the free energy of *CO desorption is much higher than that of the dimerization reaction over the OD-Cu, which indicates that *CO intermediates tend to dimerize, leading to C_2+_ products.

## 4 Conclusions

This work demonstrated interesting results on the production of alcohol (i.e. ethanol and methanol) from the conversion of CO_2_ (via electrocatalytic and thermocatalytic routes) over a Cu/ZnO catalyst prepared by the low-temperature oxidation of Cu NPs (to form Cu_2_O) and its mixing with ZnO crystalline powder.

The role of ZnO and the influence of different applied potentials on the Cu-based catalyst restructuration, and its electrocatalytic activity towards alcohol production, was studied in a liquid-phase configuration. We found that the presence of ZnO in the CZ calc fresh sample has a role in stabilizing superficial Cu^+1^ during the catalyst restructuration, which is correlated to a Zn and O enrichment with an amorphous ZnO matrix. ZnO induced a higher CO productivity on the Cu/ZnO-based electrode than on the Cu one, which increased the local CO concentration on the Cu active sites and thus, *CO surface coverage, leading to an enhanced C–C coupling and ethanol production. Moreover, the high presence of Cu^1+^ + Cu^0^ mixtures at the CZ catalyst surface was directly correlated to the ethanol production, being the main active site in this tandem catalyst for the further CO reduction to C_2+_ alcohols. Hence, an improved selectivity towards alcohol formation (approximately 8% FE_EtOH_ and 2% FE_1PrOH_) was obtained with the Cu/ZnO catalyst in contrast to the bare calcined copper (Cu calc). These results open the way for looking forward an optimal ZnO loading for achieving a suitable *CO surface coverage and tuning the CuO_*x*_ surface properties after the catalyst reconstruction. Future experimental activities in a more concentrated CO_2_ media, like in a gas diffusion electrode (GDE) cell configuration, should be exploited to determine the real potential selectivity and stability of these calcined Cu nanoparticles in an optimized mixture with ZnO, while avoiding the influence of mass-transfer limitations that hinder the CO_2_ conversion in the present case.

The TC test conducted on the Cu/ZnO catalyst demonstrated that, according to the literature, the physical mixture of Cu NPs and ZnO exhibited a synergistic effect in hydrogenating CO_2_ with respect to pure Cu-based catalysts. Methanol and CO were the only products obtained from the TC CO_2_ conversion; the methanol productivity increased from 0.21 mmol·g_cat_^–1^·h^–1^ at 200 °C to 1.375 mmol·g_cat_^–1^·h^–1^ at 300 °C with methanol selectivity that decreases from 100% at 200 °C to 23% at 300 °C. Ex situ XRD analysis demonstrates that under TC conditions, the CZ catalyst is transformed to a mixture of metallic Cu and crystalline ZnO, which deactivates because of the sintering of the particles. This phenomenon was not evidenced in the tested CZ electrodes, where the catalyst was reconstructed under less-intensive operative conditions favoring the formation of Cu^+1^. Thus, it was envisioned that the lower binding energy of *CO at the Cu^0^ catalyst surface in the TC process than in the Cu^1+^ present in the EC one leads to preferential CO production in the TC system and its further hydrogenation to methanol because of more favorable kinetic conditions than in the EC case.

Our results confirm that a good catalyst for the TC CO_2_ hydrogenation can also be promising for the EC CO_2_ conversion to alcohols. Therefore, the strategies developed in the TC field to enhance the catalyst activity and selectivity can also be exploited in the less energy-intensive CO_2_ electrocatalytic conversion process. Viceversa, the current knowledge on CO_2_R electrocatalyst reconstruction leading to *CO dimerization could be of inspiration for developing new TC systems, leading to the production of C_2+_ products.

## Supporting Information Available

XRD analyses of fresh and tested electrodes; productivity, yield, Faradaic efficiency, and CO_2_ conversion performances of the Cu calc and CZ calc catalysts at the studied applied potentials; LSV for Cu calc and CZ calc catalysts, deposited on a carbon paper support to demonstrate the reproducibility of electrocatalytic activity; XRD patterns of fresh and thermocatalytic aged CZ calc catalysts; elemental surface composition of Cu calc and CZ calc electrodes before and after testing; and formulas used to calculate the relative amount of Cu species (PDF)
