# Supplementary material for: CO2 Conversion to Alcohols over Cu/ZnO Catalysts: Prospective Synergies between Electrocatalytic and Thermocatalytic Routes
Source: ACS Appl Mater Interfaces. 2021 Dec 29;14(1):517–30. doi: 10.1021/acsami.1c15871 (PMC8762640; doi:10.1021/acsami.1c15871)
Supplement: Supplementary file 1 — am1c15871_si_001.pdf [file am1c15871_si_001.pdf]

Supporting Information

CO_2 conversion to alcohols over Cu/ZnO catalysts: prospective synergies between electrocatalytic and thermocatalytic routes

Hilmar Guzmán,[a,b] Fabio Salomone,[a] Samir Bensaid,[a] Micaela Castellino, [a] Nunzio Russo,[a] Simelys Hernández [a,b,]*

[a] *CREST group, Department of Applied Science and Technology (DISAT), Politecnico di Torino, C.so Duca degli Abruzzi, 24, 10129, Turin, Italy.*

[b] *IIT – Istituto Italiano di Tecnologia, Via Livorno, 60, 10144, Turin, Italy.*

* *Corresponding author. E-mail address:* simelys.hernandez@polito.it

Summary:

S1. XRD analyses of fresh and tested electrodes 2

S2. The total products distribution from the EC CO_2R 3

S3. Reproducibility of electrocatalytic activity of catalysts 5

S4. XRD analysis of TC tested CZ calc catalyst 6

S5. Elemental surface composition of electrodes obtained from XPS analysis. 7

S6. Reference. 8

S1. XRD analyses of fresh and tested electrodes

The analyses were carried out by using an automatic Philips X'Pert θ/2θ powder diffractometer with Bragg-Brentano geometry, using X Cu Kα radiation with λ=1.5416 Å and power 1.6 kW. The angular range used is from 5 to 90 ° (2θ) with steps of 0.02 ° (2θ) and an acquisition time of 15 seconds per step. The search for the inorganic phases identified in the various samples was performed using the Hanawalt research method on the PDF-2 data set (Powder Diffraction File, ICDD). The quantitative analysis of the identified species was completed by applying the Rietveld method implemented in the Topas software.

The Teflon $(C_2F_4)n$ and Graphite reflection peaks relate to the support the catalysts are deposited. In Table S1, you can see the relative abundance of copper and its oxides as well as of ZnO. It is noted that in the CZ sample that nominally contains zinc oxide when passing from fresh to tested, zincite is no longer detectable in bulk. In addition, there is not any trace of copper oxides in the CZ calc tested sample. Instead, in the Cu calc tested sample, the presence of Cu^{1+} (Cuprite) is recognized. That amount of Cu^{1+} is less than in the fresh electrode.

This apparent discrepancy between the results obtained by XPS and those obtained here by XRD on the fresh and tested samples can be explained by bearing in mind the different areas of investigation linked to the two analytical techniques: surface for XPS, bulk for XRD.

Table S1**.** Bulk composition (wt%) obtained from XRD spectra and Rietveld method of electrodes before and after testing under CO_2 electroreduction conditions at -1.4V vs RHE.

Electrode	(C_2F_4) Teflon	Graphite	Cu	Cu_2O	CuO	ZnO
Cu calc Fresh	8.6	1.0	12.0	78.4	-	-
Cu calc Tested	14.3	2.7	50.0	33.0	-	-
CZ calc Fresh	4.0	1.0	7.1	78.2	-	7.1
CZ calc Tested	13.3	5.7	81.0	-	-	-

S2. The total products distribution from the EC CO_2R

The EC CO_2R was carried out by applying a constant potential for 2 hours at ambient conditions. The CO_2 flow rate was set at 8.86 NmL min^{-1}. The selectivity of each catalyst material at the corresponding applied potential is listed in the tables below.

Table S2**.** Productivity, yield and CO_2 conversion performances of the Cu calc and CZ calc catalysts (at -1.40 V vs RHE).

Electrode	Productivity, mmol g_{cat}^{-1}. h^{-1}								Yield, mmol g_{cat}^{-1}. $g_{CO2}INLET^{-1}$								CO_2 Conversion, %
	H_2	CO	CH_4	C_3H_6O	CH_3OH	Ethanol	2-propanol	Formate	H_2	CO	CH_4	C_3H_6O	CH_3OH	Ethanol	2-propanol	Formate	
Cu calc	492.96	20.84	1.06	0.06	0.12	3.59	0.06	72.49	257.50	10.89	0.55	0.03	0.06	1.88	0.03	37.87	0.29
CZ calc	401.30	41.29	0.83	0.06	0.09	5.27	-	6.85	209.62	21.57	0.43	0.03	0.05	2.75	-	3.57	0.34

Table S3**.** Faradaic efficiency performances of the Cu calc and CZ calc catalysts (at -1.40 V vs RHE).

Electrode	Faradaic efficiency, %							
	H_2	CO	CH_4	C_3H_6O	CH_3OH	Ethanol	2-Propanol	Formate
Cu calc	57.72	2.44	0.50	3.00	2.15	2.52	0.06	9.00
CZ calc	76.92	7.91	0.64	0.09	0.44	8.00	-	1.31

Table S4**.** Productivity, yield and CO_2 conversion performances of the CZ calc catalyst (at different applied potentials).

V vs RHE	Productivity, mmol g_{cat}^{-1}. h^{-1}									Yield, mmol g_{cat}^{-1}. $g_{CO2}INLET^{-1}$									CO_2 Conv., %
	H_2	CO	CH_4	C_3H_6O	CH_3OH	Ethanol	2-propanol	1-propanol	Formate	H_2	CO	CH_4	C_3H_6O	CH_3OH	Ethanol	2-propanol	1-propanol	Formate	
-0.56	27.34	6.82	2.95	0.01	0.15	0.01	0.001	-	0.49	14.28	3.56	1.54	0.00	0.08	0.01	0.001	-	0.26	0.06
-0.69	21.41	5.78	0.56	0.00	0.00	0.08	0.002	-	1.77	11.18	3.02	0.29	0.00	0.00	0.04	0.001	-	0.92	0.04
-0.80	149.82	41.30	-	0.03	0.10	1.16	-	0.27	5.72	78.26	21.57	-	0.02	0.05	0.61	-	0.14	2.99	0.23
-1.40	401.30	41.29	0.83	0.06	0.09	5.27	-	-	6.85	209.62	21.57	0.43	0.03	0.05	2.75	-	-	3.57	0.34

Table S5. Faradaic efficiency performances of the CZ calc catalyst (at different applied potentials).

Potential, V vs RHE	Faradaic efficiency, %								
	H_2	CO	CH_4	C_3H_6O	CH_3OH	Ethanol	2-Propanol	1-Propanol	Formate
-0.56	55.00	15.93	5.02	0.18	2.11	2.76	0.03	-	2.35
-0.69	45.00	9.75	3.79	0.02	0.01	0.75	0.03	-	8.00
-0.80	64.63	17.82	--	0.13	0.17	4.01	--	1.5	3.29
-1.40	76.92	7.91	0.64	0.09	0.44	8.00	--	-	1.31

S3. Reproducibility of electrocatalytic activity of catalysts

Figure S1 demonstrates that the electrocatalytic activity of the here studied materials is reproducible.

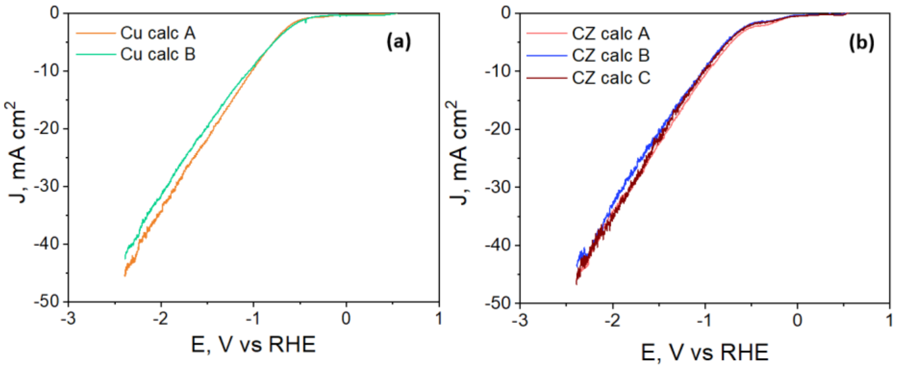

Figure S1. Linear sweep voltammetry (LSV) for Cu calc (a) and CZ calc b) catalysts, deposited in a carbon paper support. The test was carried out in a CO_2-purged 0.1 M $KHCO_3$ electrolyte (scan rate: 5 mV s^{-1}) with a catalyst loading of 1.5 mg cm^{-2} in a three-electrodes electrochemical cell.

S4. XRD analysis of TC tested CZ calc catalyst

Figure S2 shows the patterns of fresh and TC aged CZ calc catalysts. It is worth noting that the fresh CZ calc catalysts are composed of Cu, Cu_2O and ZnO. In fact, it is a physical mixture of Cu NPs and ZnO powder. On the contrary, the aged CZ calc catalysts are composed of metallic Cu and ZnO because the Cu_2O was reduced during the TC tests. In addition, the Cu crystallite size increased from 31 nm to 61 nm, while the ZnO crystallite size increased from 15 nm to 25 nm.

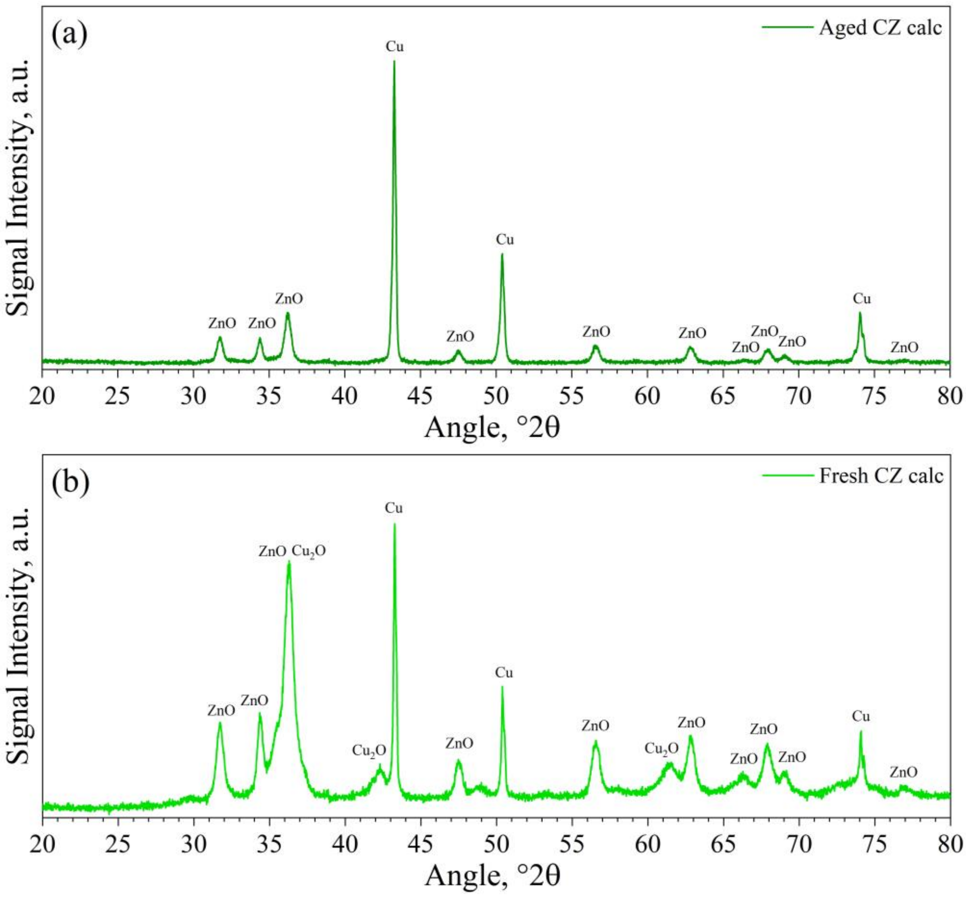

Figure S2. XRD patterns of (a) TC aged CZ calc catalyst and (b) fresh CZ calc catalyst.

S5. Elemental surface composition of electrodes obtained from XPS analysis.

The superficial composition of the electrodes before and after the tests was calculated from the survey XPS spectra and is reported in Table S6. The Cu calc electrode is characterized by Cu species on its surface highly covered by the Nafion binder, as observed by the high quantities of F and S atoms in both fresh and tested samples. Besides, the high amount of C could be mainly ascribed to the multi-walled carbon nanotubes (MWCNT) used to increase the catalytic layer conductivity. Instead, the superficial coverage by Nafion of the CZ calc catalyst in the fresh electrode is about 34% lower than in the Cu fresh one, while after testing the surface of this electrode has a much lower amount of Nafion (F and S are below the detection level of the XPS) and is highly enriched of Zn, Cu and O elements. The 46% lower amount of C in the CZ calc tested samples indicates that, after testing, the MWCNT were provably recovered by the reconstructed catalytic layer.

Table S6**.** Surface elemental composition (at.%) obtained from XPS analyses of the electrodes before and after testing under CO_2 electroreduction conditions at -1.4V vs RHE.

Electrode	Cu	O	C	Zn	K	F	S	Cu/O	Zn/Cu
Cu calc Fresh	1.9	4.8	62.9	-	-	29.8	0.6	0.4	
Cu calc Tested	0.9	7.4	65.4	-	1.7	24.2	0.4	0.1	
CZ calc Fresh	1.6	7.3	70.1	1.3	-	19.5	-	0.2	0.8
CZ calc Tested	3.8	43.1	37.9	13	2.1	-	-	0.1	3.4

The precise amount of the copper species (having different oxidation states) on each sample was estimated through the method developed by M. Biesinger and co-workers.[1] The formulas used to calculate the relative amount of Cu species are listed below:

$$\%\big(Cu(0) + Cu(I)\big) = (A - k \cdot B)/(A + B) \cdot 100 \qquad (1)$$

$$\%\big(Cu(II)\big) = B(1 + k)/(A + B) \cdot 100 \qquad (2)$$

where:

- A is the total area of the main peak $Cu2p_{3/2}$;
- B is the total area of shake-up satellite peak;
- k is a constant value related to Cu reference samples.

The results are reported in Figures 6 and 7 and are discussed in the manuscript.

S6. Reference.

1 M. C. Biesinger, L. W. M. Lau, A. R. Gerson and R. S. C. Smart, *Appl. Surf. Sci.*, 2010, **257**, 887–898.
